# Production of Non-Ribosomal Peptide Synthetase (NRPS)- Dependent Siderophore by Aeromonas Isolates

**DOI:** 10.7508/ibj.2016.04.008

**Published:** 2016

**Authors:** Ramasamy Amsaveni, Muthusamy Sureshkumar, Arthanari Aravinth, Joseph Reshma Mary, Govindasami Vivekanandhan

**Affiliations:** 1 Department of Biotechnology, Kongunadu Arts and Science College, Coimbatore-641029, Tamilnadu, India; 2 K.S.R. College of Arts and Science, Tiruchengode-637215, Tamilnadu, India; 3 Farmer’s Bio-Fertilizers and Organics, 461, Sri Ragavendra Gardens, G.N. Mills Post, Coimbatore-641029, Tamilnadu, India

**Keywords:** *Aeromonas*, Siderophores, Hydroxamate

## Abstract

**Background:**

*Aeromonas* species are Gram-negative ubiquitous bacteria, facultative anaerobic rods that infect both invertebrates and vertebrates. Various fish species develop hemorrhagic disease and furunculosis due to *Aeromonas* spp. *Aeromonas* strains generate certain active compounds such as siderophores, which are the final products of non-ribosomal peptide synthetase (NRPS) activity. The present study attempted to investigate the prevalence of *Aeromonas* isolates in marketed fish sources. We also examined the siderophore production ability of these isolates.

**Methods:**

Among the molecular tools, 16S rRNA analysis was used to identify *Aeromonas* species and their epidemiological distributions. The hemolytic activity of the strains and biochemical assays were used to confirm the identity of the isolates. We also determined the chemical nature of siderophores in these strains.

**Results:**

A total of seven *Aeromonas* isolates obtained from fish were included to determine the siderophore production. Of 7 isolates, 4 produced siderophore, and their chemical nature was also determined. The siderophore produced by *Aeromonas* was invariably found to be of hydroxamate. Four *Aeromonas* isolates were selected for PCR identification of NRPS-encoding gene. The conserved sequence was present in all four selected isolates. Furthermore, siderophores were qualitatively tested for their antibacterial activity against pathogenic bacteria and a significant level of inhibitory activity was observed in siderophores from the four isolates.

**Conclusion:**

Our results showed the ability of the isolated strains in production of siderophores with a high level of activity against *Salmonella paratyphi*. These siderophores could find applications in biomedical industries.

## INTRODUCTION

*Aeromonas* spp. was thought to be an opportunistic pathogen in immunocompromized humans. The species belongs to the family Aeromonadaceae and has a broad host spectrum, with both cold- and warm-blooded organisms, including human being[1]. *Aeromonas* spp. are catalase- and oxidase-positive bacteria and reduce nitrate to nitrite, as well as they show large zones of hemolysis around colonies on blood agar[2]. In fish, these bacteria cause hemorrhagic septicemia, fin rot, soft tissue rot and furunculosis[3]. A variety of extracellular virulence factors such as enterotoxins, cytotoxins, hemolysins, erolysins, proteases and hemagglutinins produced by *A. hydrophila* support their epidemiological associations[4]. Apart from extracellular virulent proteins, *Aeromonas* also produces the catecholate siderophores, an iron-binding protein either amonabactin or enterobactin that are considered as virulence factors[5]. As iron is an essential element for the growth of almost all known organisms, many saprophytic and pathogenic microorganisms have evolved some methods of sequestering iron from their environment, which is in the form of insoluble ferric oxide/hydroxide complexes. One way of achieving this goal is the production of small molecules with high affinity for ferric iron, known as siderophores, whose biosynthesis is induced by intracellular iron deficiency and are secreted into the environment to scavenge iron.

Non-ribosomal peptides (NRPs) are a class of secondary metabolites that are usually produced by microorganisms such as bacteria and fungi. Unlike the polypeptides, which are synthesized on ribosomes, NRPs are produced by NRPs, which are considered as preassembled, modular molecular factories. Also, contrary to the ribosome, which is fed by an mRNA code and can make an arbitrary sequence of peptides, an NRPs does not accept a code and is preset to make one peptide. As a class, NRPs can make a wider diversity of peptides than ribosomes. They are structurally a very diverse family of natural products with an extremely broad range of biological activities and pharmacological properties. NRPs are often toxins, siderophores or pigments. There is an enormous scope for the application of microbial siderophores for the sustainability of humans, animals and plants[6].

Hydroxamate siderophores are present in soil at concentrations high enough to be absorbed by plant roots. It has been reported that hydroxamate siderophores exist in various soils, and they are also produced in aquatic environments[7]. Siderophore synthesis, their structure, properties and applications have been studied in many terrestrial microorganisms[8-12].

Based on various aspects and beneficial applications of siderophores in all sectors, the current research has been carried out to screen *Aeromonas* spp. for production of siderophores and investigate the nature and possible biological activities of these compounds.

## MATERIALS AND METHODS

### Sample collection and processing

Collection and processing of marine fish specimens for the isolation of *Aeromonas* were performed as reported earlier[13]. Briefly, fish samples were collected randomly from fish retail outlets in and around Coimbatore City, Tamilnadu, India and transported to the laboratory in less than an hour. Various organs such as body surface, gill and intestine of the fish were swabbed using sterile cotton swabs and enriched in alkaline peptone water overnight. The enriched cultures were streaked on starch ampicillin agar medium and incubated at 28°C for 24 h. Colonies with yellow to honey color were selected and used for further examination.

### Identification of Aeromonas

Pure cultures were identified as *Aeromonas* based on biochemical reactions[14] and were subjected to the amplification of 16S rRNA gene. Aero16S F (5’CAGAAGAAGCACCGGCTAAC-3’) and Aero16S R (5’TTACCTTGTTACGACTTCAC-3’) primers were used with the following PCR conditions, and the gene was amplified in a thermal cycler (Eppendorf). Polymerase chain reaction was performed as initial heating at 94°C for 5 min, 30 cycles of denaturation at 94°C for 1 min, annealing at 55°C for 1 min, extension at 72°C for 1 min, followed by a final extension at 72°C for 10 min. The PCR products were then separated on 1% agarose gel.

### The hemolytic activity of Aeromonas isolates

The hemolytic activity of the isolates was determined by using blood agar plates containing 5% (v/v) anticoagulant-added blood. The zone of hemolysis around the colonies on the plates after 24-h incubation will be the positive result.

### Siderophore production and qualitative detection of siderophores

#### Ferric chloride (FeCl_3_) test

All the seven isolates were used for the analysis of siderophore production by FeCl3 method[15]. About 1 mL 2% FeCl_3_ solution was added to 1 mL of culture supernatant. The development of orange or red-brown color of the supernatant indicated the presence of siderophore.

#### Chrome azurol sulfonate (CAS) assay[16]

Culture supernatant (1 mL) was added to 1 mL CAS assay solution. Then the supernatant was examined for the change of color from blue to orange or pale reddish brown, which showed the presence of siderophore.

### Chemical assays for determination of siderophores identity

All the qualitatively tested isolates were subjected to the analysis of their chemical groups by performing the following tests.

#### Tetrazolium salt test[16]

The test was carried out by the addition of 1-2 drops of 2 N NaOH and 0.1 mL of the test culture supernatant to a pinch of tetrazolium salt. The appearance of a red to deep-red color confirmed the presence of hydroxamate siderophores.

#### Vogel’s test[17]

In this test, 1 drop of phenolphthalein was added to 3 drops of 2 N NaOH. Sterile deionized water was then added to the mixture until the appearance of a light pink color. Disappearance of color by the addition of culture supernatant indicated the presence of siderophores with carboxylate nature.

#### Arnow’s assay[18]

At first, 0.5 mL reagent containing nitrite molybdate with 0.1 mL of 2 N NaOH was added to 1 mL culture filtrate and 0.1 mL of 1 N HCl. Next, the volume was made up to 5 mL. The absorbanceat 515 nm in UV-VIS spectrophotometer indicates the catecholate nature of siderophores.

#### 2% ferric chloride test

Hydroxamate, catecholate and carboxylate or other siderophores capable of forming stable iron complexes at low pH could be detected with the addition of 2.5 mL 2% FeCl_3_ to 0.5 mL culture supernatant. The nature of siderophores was ascertained by the examination of their OD (absorption) peak between 420-450 nm (hydroxamate), 495 nm (ferric catecholate), 250, 320 and 210 nm (catecholate) and 190-280 nm (carboxylate) using a UV-visible spectrophotometer. A peak between 420 and 450 nm of ferrated siderophores indicated its hydroxamate nature.

### PCR amplification of siderophore-related non-ribosomal peptide synthetase gene

Siderophore encoding NRPS gene was amplified by specific primers: F1 (5’ TTGTTGACCTATGGCGAG CTGGAG 3’) and R1 (5’ CGGCACCATGTATTCG GGCAG 3’) using a thermal cycler with the following PCR conditions: Initial heating at 94°C for 5 min, 30 cycles of denaturation at 94°C for 30 s, annealing at 56°C for 30 s and extension at 72°C for 1 min, followed by a final extension at 72°C for 5 min.

### Antimicrobial activity of siderophores

The antimicrobial activity of the siderophores produced by *Aeromonas* isolates was determined by agar well diffusion method[19]. The pathogenic bacterial isolates used were *E. coli*, *Klebsiella* sp., *Proteus* sp., *Pseudomonas* sp., *Salmonella typhi*, *S*. *paratyphi*, *Vibrio* sp., *Bacillus* sp., *Staphylococcus* sp. and *Streptococcus* sp. The results were expressed as the average diameter of inhibition zones around the wells.

## RESULTS

### Isolation and identification of Aeromonas

Marine fish samples were processed. Based on biochemical profiling and 16S rRNA (1050 bp) gene amplification, yellow to honey colored colonies on starch ampicillin agar plates, bacterial colonies were recovered and identified as *Aeromonas*. Eight *Aeromonas* isolates were obtained from 26 fish samples. It was observed that all the isolates were capable of producing β-hemolysin.

### Hemolytic activity and siderophore production of Aeromonas isolates

**Fig. 1:**
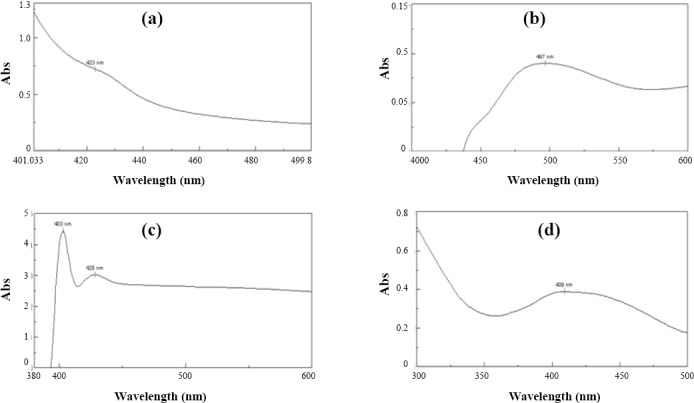
Spectrophotometric assay showing peaks for hydroxamate type of siderophores. a, b, c, and d show the spectrum of siderophore produced by A5, A7, A19 and A27, respectively. A, *Aeromonas*; Abs, absorbance

FeCl_3_ and CAS assays indicated that 4 out of 8 isolates (A5, A7, A19 and A27) produced siderophore. Of 4 isolates, 3 (A5, A19 and A27) produced hydroxamate-type siderophore, which was confirmed qualitatively by tetrazolium salt test. Using Neilands’ spectrophotometric assay, the peaks of the isolates were recorded at 423 nm, 428 nm and 409 nm, respectively (Fig. 1a, Fig. 1c and d)[20]. However, the other isolate, A7, produced a catecholate type, which was confirmed by Arnow’s test. The result indicated a light wine color, and a peak was recorded at 497 nm (Fig. 1b). The isolate A19 produced hydroxamate-type siderophore as well as catecholate type siderophore, where the peak was observed at 403 nm (Fig. 1c). None of the isolates produced carboxylate-type siderophore as Vogel’s test result showed stable pink colors, indicating the negative results for carboxylate-type siderophore.

### Identification of non-ribosomal peptides-encoding gene by the PCR method

Selected isolates of *Aeromonas* (A5, A7, A19 and A27) were subjected to the identification of NRPS-encoding gene by the PCR method using degenerative primers. The conserved sequence of NRPS gene was found to be present in all the isolates.

### Antibacterial activity of siderophores

**Fig. 2:**
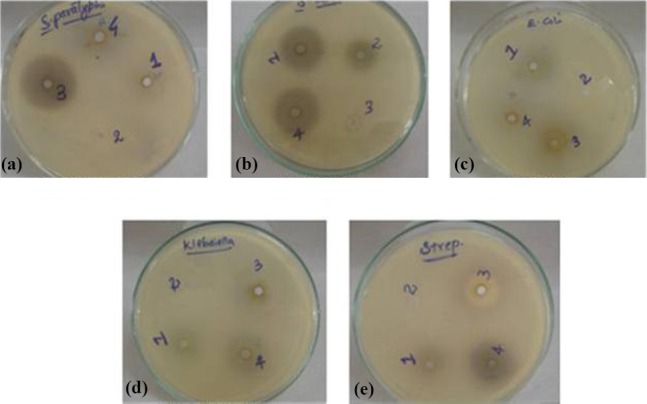
Antibacterial activity of crude siderophores. a) *Salmonella paratyphi*; b) *Salmonella typhi*; c) *Escherichia coli*; d) *Klebsiella* sp.; e) *Staphylococcus* sp, showing the inhibitory zones formed by crude siderophore extracts against bacterial pathogens. Note: 1, A5; 2, A7; 3, A19; 4, A27; A, *Aeromonas*

Siderophores were qualitatively tested for their antibacterial activity at 37°C for 24 h. The crude siderophore extracts of all the four *Aeromonas* isolates showed a moderate level of antibacterial activity (Table 1). There was the highest level of antibacterial activity against *Salmonella paratyphi*. Siderophore extracts of the isolates A19 and A27 inhibited *Klebsiella, E. coli*, *S. typhi*, *S. paratyphi* and *Streptococcus* sp. Siderophore of A27 isolate exhibited a maximum inhibition zone of 18 mm against *S. paratyphi*. *Proteus* sp.*, Pseudomonas* sp.*, Vibrio* sp., *Bacillus* sp., and *Staphylococcus* were found to be resistant to the siderophores of all the four isolates (Fig. 2).

**Table 1:** Antibacterial activity of crude siderophores

Serial No.	Bacterial pathogens	Siderophores of *Aeromonas* zone of inhibition (mm)			

		A5	A7	A19	A27
1	*E. coli*	14.0	0	12.0	8.0
2	*Klebsiella* sp.	8.0	0	10	10.0
3	*Proteus* sp.	0	0	0	0
4	*Pseudomonas* sp.	0	0	0	0
5	*S. typhi*	16.0	12.0	14.0	14.0
6	*S. paratyphi*	16.0	0	18.0	16.0
7	*Vibrio* sp.	0	0	0	0
8	*Bacillus* sp.	0	0	0	0
9	*Staphylococcus* sp.	0	0	0	0
10	*Streptococcus* sp.	8.0	0	12.0	14.0

A, *Aeromonas*

## DISCUSSION

Fishery products are of great importance for human nutrition worldwide and can act as a source of food-borne pathogens[21]. The incidence of microbial pathogens, especially those of bacterial origin, is one of the most significant factors affecting fish culture[22]. About 28% incidence of *A. hydrophila* has been reported in fishes from retail outlets of New Zealand[23], and 90% maximum incidence has been recorded in marketed fish at Coimbatore, South India[24]. In the present investigation, the prevalence of *Aeromonas* in various fish indicated that 31% of the fish samples contained *Aeromonas* isolates.

*Aeromonas* secretes several extracellular proteins, including enterotoxin, hemolysin and erolysin that are associated with the bacterial virulence[25]. The production of hemolytic toxins has been regarded as strong evidence of pathogenic potential in aeromonas[26]. All the eight selected isolates showed β-hemolytic activity. We also observed stronger hemolytic activity on agar plates, which confirms the presence of virulent *Aeromonas* isolates in fish.

Siderophores are common products of aerobic and facultative anaerobic bacteria and fungi. In the current study, the production ability of hydroxamate and catecholate types of siderophore was observed in the *Aeromonas* isolates obtained from marine fish. It has been suggested that quantitative variation in siderophore production is related to space, time, environment and organisms concerned[27]. In the present study, the production of siderophore was primarily confirmed by positive FeCl_3_ test. Positive results for Neilands’ spectrometric assay showed a maximum absorption at 430 nm, indicating hydroxamate nature of siderophores. Mixed type of siderophores (hydroxamate and catecholate) produced by Pseudomonas has also been reported [28].

The present study investigated the antibacterial activity of the crude siderophore extracts produced by Aeromonas isolates. The results indicated a significant level of inhibitory activity in siderophores from four isolates against pathogenic bacteria. We identified the prevalence of *Aeromonas* isolates in marketed fish sources. About 31% of the fish samples were found to be contaminated with pathogenic *Aeromonas* isolates. It is an important threat to the people consuming contaminated fish and other sea foods. The crude siderophores included in this study showed antimicrobial activity against pathogenic bacterial isolates. Further studies are required to examine a wide range of clinical pathogens.
